# Analysis of the Spectral Characteristics of Pure Moxa Stick Burning by Hyperspectral Imaging and Fourier Transform Infrared Spectroscopy

**DOI:** 10.1155/2016/1057878

**Published:** 2016-09-19

**Authors:** Xiao-jing Song, Shu-you Wang, Yin-long Li, Dong Zhang

**Affiliations:** Institute of Acupuncture and Moxibustion, China Academy of Chinese Medical Sciences, No. 16, Nanxiaojie, Dongzhimen, Beijing 100700, China

## Abstract

The objective of this study was to investigate the spectra characteristics (SC) at wavelengths of 400~1000 nm and 2.5~15.5 *μ*m of pure moxa stick (MS) during its 25-minute burning process using new spectral imaging techniques. Spectral images were collected for the burning pure MS at 5, 10, 15, 20, and 25 min using hyperspectral imaging (HSI) and Fourier transform infrared spectroscopy (FTIR) for the first time. The results showed that, at wavelengths of 400~1000 nm, the spectral range of the cross section of MS burning was 750~980 nm; the peak position was 860 nm. At wavelengths of 2.5~15.5 *μ*m, the spectral range of the cross section of MS burning was 3.0~4.0 *μ*m; the peak position was approximately 3.5 *μ*m. The radiation spectra of MS burning include litter red and amount of infrared (but mainly near infrared) wavelengths. The temperature, blood perfusion, and oxygen saturation increase of Shenshu (BL23) after moxibustion radiation were observed too. According to mechanism of photobiological effects and moxibustion biological effects, it was inferred that moxibustion effects should be linked with moxibustion SC. This study provided new data and means for physical properties of moxibustion research.

## 1. Introduction

The term “spectrum” refers to the range of colors (according to the wavelength or frequency) observed when white light is separated by dispersion through a prism or grating. Spectroscopy is an analytical method for measuring the spectrum based on the inherent relation between the irradiation and absorption of electromagnetic radiation of matter and the composition and structure of matter [1, 2]. In recent years, spectroscopy has received increasingly more applications in the life sciences for detecting and determining drug ingredients [3–5], pathological diagnosis [6, 7], detection and diagnosis of diseases, and so forth [8–10]. However, there are very few reports of the application of spectroscopy in moxibustion [11, 12]. In the present study, to expand the application of spectral imaging techniques to moxibustion, we observed and analyzed the spectra characteristics (SC) of moxa stick (MS) burning using hyperspectral imaging (HSI) and Fourier transform infrared spectroscopy (FTIR).

Over thousands of years, moxibustion has been an important component of traditional Chinese medicine. MS has been chosen as the main herb for moxibustion in Chinese medicine; the effective substances and energy produced by the burning of MS can stimulate acupoints and organs, thereby achieving the effect of treating diseases and promoting health. The ancients believed that moxibustion can warm channels to dispel coldness, promote blood circulation to remove meridian obstruction, dissipate mass and relieve pain, compensate weakness and yang deficiency, and so forth [13]. Modern research has also shown that moxibustion can promote the metabolism of organisms, effectively improve the immunity and endocrine function of organisms, and treat systemic diseases involving circulation, digestion, respiration, immunity, and so forth [14–17]. With the development of science and technology, people have begun to study the mechanism of moxibustion from the standpoint of biology, chemistry, and physics [18–20]. MS produces a biological effect through burning. Therefore, observing and analyzing the physical characteristics of MS burning is one important research direction to investigate the mechanism of moxibustion. In present study, HSI and FTIR were used to observe the SC of MS burning at wavelengths of 400~1000 nm and 2.5~15.5 *μ*m. And the temperature, blood perfusion (BP), and oxygen saturation (SaO_2_) changes of Shenshu (BL23) acupoint skin were observed before and after moxibustion to discuss the relationship between SC and effect of moxibustion.

## 2. Materials and Methods

### 2.1. Detection Principle of HSI and FTIR

**Figure 1:**
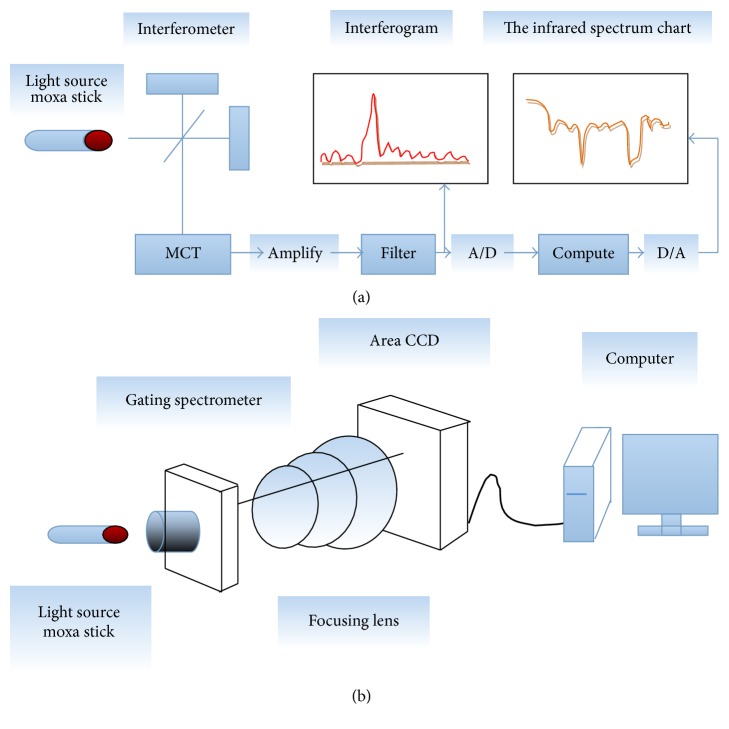
Schematics of the principle of hyperspectral imaging and Fourier transform infrared spectroscopy. (a) is the schematic for HSI data collection and recording, while (b) is the schematic for FTIR data collection and recording.

HSI is an imaging technique based on multiple imaging to continuously image targets and utilizes the spectral coverage of hundreds of wavelengths of an imaging spectroradiometer at a wavelength of 400~1000 nm; spectral information is gained while obtaining the spatial imaging of targets. The hardware components of HSI mainly include a spectral camera (imaging spectroradiometer + CCD), system software, and a computer [21]. The principle of HSI data collection and recording of MS burning is presented in Figure 1(a). FTIR is an interference spectroscopy based on light interference. It mainly consists of a light source, interferometer, detector, computer, and recording system. The experimentally obtained raw spectrum is converted to a wavelength-based or wavenumber-based spectrum through fast Fourier transformation using a computer [22]. The principles used to collect FTIR data and record MS burning are presented in Figure 1(b).

### 2.2. HSI Data Collection and Analysis of MS Burning

The temperature of the experimental environment was 25 ± 1°C, and the relative humidity was 30~60%. The indoor and outdoor ventilation was isolated. The experiment was performed in a dark room with no light source. The spectral wavelength range was 400~1000 nm, the spectral resolution was 10 nm, and the exposure time was 1 s; in addition, the instrument's camera lens was capped for data collection, calibration, and storage. High-quality pure MS (Beijing Zhongyan Taihe Medicine Limited, Beijing) was burned, and the burning section was placed in front of the HSI camera lens and 15 cm from its center until full burning. The optimal location was observed and determined by the in situ monitoring function included in the computer's HSI data collection software. The radiation spectrum was collected and recorded at 5 min, 10 min, 15 min, 20 min, and 25 min during the burning process. The instrument was connected to the computer, and PHySpec image application software was used to collect, process, and store the HSI spectra. PHySpec software was used to extract 5 × 5 mm HSI images and to display the mean spectrum of moxibustion at different times. Then, the spectral range and the intensity of absorption and reflectance were analyzed to compare changes in the spectra at different time points.

### 2.3. FTIR Data Collection and Analysis of MS Burning

The experimental environment for FTIR data collection of MS burning was the same as that for HIS data collection. High-quality pure MS was burned, and the burning section was placed in front of the light-receiving window of the FTIR instrument and 15 cm from its center until full burning. Fourier transformation was used to convert the IR spectra of the burning MS collected by the FTIR instrument. The sample scanning time was 1; background scanning time was 1 s; resolution was 4.0; final format was single beam; scanning range was 4000~400 cm^−1^; spectral gaining was automatic; optical gating was 100; light source was external light path. The IR radiation spectrum was collected and recorded at 5 min, 10 min, 15 min, 20 min, and 25 min during the burning process. OMNIC 7.0 software was used to analyze the spectral range (SR) and the relative radiation intensity (RRI) of the emitted single beam spectra and to compare the spectral changes at different time points.

### 2.4. Temperature, BP, and SaO_2_ Measurement of BL23 Acupoint Skin

Ten healthy adult volunteers without skin disease (4 females and 6 males: ages ranged from 22 to 25 years) were recruited in this study. Prior to participation, all subjects provided written informed consent. All experimental procedures were approved by the Ethical Committee of China Academy of Medical Sciences and conducted in accordance with the international accepted principles. The subjects waited quietly for 15 min in the detection environment which was 25 ± 1°C, with relative humidity of 30–60% and no ventilation and no solar radiation. The burning MS was conducted vertically above 3.5 cm of the right BL23 acupoint for 10 min. It should be subjected to occurrence of flushing and tolerance of the subject. Before moxibustion, at 10 minutes after moxibustion, at 15 minutes after stopping moxibustion, and at 30 minutes after stopping moxibustion, the infrared thermogram (IRT) of waist was scanned by VARIOSCAN3021-ST infrared thermal imager (InfraTec Ltd., Germany). The temperature of right BL23 acupoint skin was analyzed by IRBIS 2.2 software. The BP image of waist was scanned by Moor-FLPI laser speckle perfusion (LSP) imager (Moor Instruments Ltd., Axminster, UK). The scanning model was the low density and 25 fps, the time interval was 1 s, exposure time was 20 ms, and 10 frames were continually scanned at each time point. The BP of right BL23 acupoint skin was analyzed by Moor-FLPI software. The pixel range of 5 × 5 mm for the acupoint in the IRT (LSP image) was selected to display the value of temperature (BP). Then, SaO_2_ of right BL23 acupoint skin was measured by ODISsey SaO_2_ monitor (ViOptix Ltd., USA).

### 2.5. Statistical Analysis

The mean differences of temperature, BP, and SaO_2_ of right BL23 acupoint skin in each group were calculated at each time point to investigate the changes in the parameters with time. One-way ANOVA with LSD test was used to test differences in each indicator between groups using SPSS 19.0 statistical software. The data were represented as $\bar{x}\pm SE$. Differences with *P* < 0.05 were considered statistically significant.

## 3. Results

### 3.1. HSI Images of MS Burning at a Wavelength of 400~1000 nm

**Figure 2:**
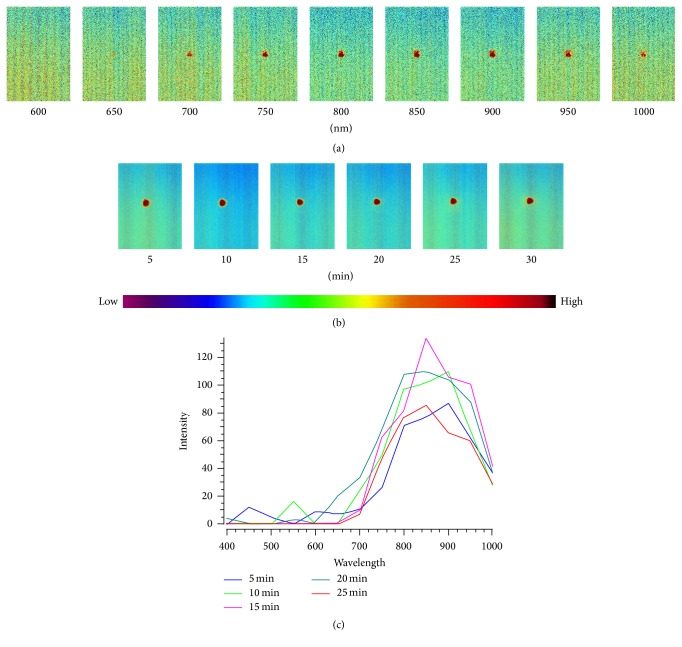
HSI of MS burning at a wavelength of 400~1000 nm. (a) is the HSI of a pure MSCS that has been burned for 5 min at wavelengths of 600, 650, 700, 750, 800, 850, 800, 950, and 1000 nm. The color depth on the HSI image represents the intensity of the reflected light. The darker the color is, the higher the intensity is and vice versa. The color code from low to high denotes the RRI from low to high. Before 650 nm, the MSCS burning did not produce imaging, while the color of MSCS burning darkened gradually after 650 nm and became lighter after 850 nm. (b) is the HSI of MSCS at different burning time points at the wavelength of 850 nm; (c) is the spectrum of MSCS at different burning time points at the wavelength of 400~1000 nm.

Sixty-one HSI images of the cross sections were collected for the pure MS that was fully burning. Nine images were chosen to present HSI images of the pure MS at different wavelengths. The color depth on the HSI image represents the intensity of the reflected light. The darker the color is, the higher the intensity is and vice versa. Under static and full burning, the color of the MS's cross section (MSCS) was the same as that of the background at the visible wavelength of 400~640 nm, indicating that the light intensity of the MSCS cannot be detected at this wavelength. After 650 nm, the burning MSCS on the image exhibited an orange color, and the color darkened with increased wavelengths. The light intensity of the MSCS increased at this wavelength. After 900 nm, the color of the MSCS became slightly lighter (Figure 2(a)).

### 3.2. Spectra Characteristics of MS Burning at a Wavelength of 400~1000 nm

The spectra of the MSCS were extracted from HSI for analysis. The ordinate represents the RRI produced from the MS burning, and the abscissa represents the wavelength. The characteristics of the HSI at different burning times are consistent (Figure 2(b)). For pure MS that was fully burned for 5 min in the static phase, the spectrum was distributed at a wavelength of 640~1000 nm, ascended at 640~820 nm, reached its peak at approximately 820 nm, and descended at 820~1000 nm. The peak position was in the range of 750~975 nm, and the RRI of the peak is 340. The characteristic spectrum was distributed at red and near infrared (mainly near infrared) wavelengths. The RRI of the peak values at different burning times was stable. The spectral intensity (SI) of MSCS fluctuated slightly during the burning (Figure 2(c)).

### 3.3. Spectral Characteristics of MS Burning at a Wavelength of 2.5~15.5 *μ*m

**Figure 3:**
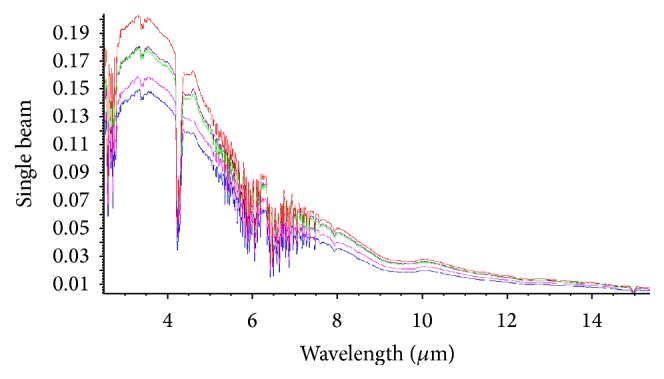
Spectra of the MS burning at a wavelength of 2.5~15.5 *μ*m at different time points. The red line denotes burning for 5 min, purple line denotes burning for 15 min, green line denotes burning for 10 min, blue line denotes burning for 20 min, and pink line denotes burning for 25 min.

For the static and full burning process, the FTIR spectra were analyzed for the MSCS at 2.5~15.5 *μ*m. The ordinate represents the RRI produced from the MS burning, and the abscissa represents the wavelength. For pure MS that was fully burned for 5 min, the spectrum ascended at 2.7~3.5 *μ*m, fluctuated slightly at 3.5~3.6 *μ*m, and descended after 3.6 *μ*m. The main shape of the IR radiation spectrum for MSCS was at 2.7~3.5 *μ*m. The PP was at approximately 3.5 *μ*m, the RRI corresponding to the peak was approximately 0.18, and the PP was in the range of 3.0~4.0 *μ*m. In addition, there was an obvious valley in the descending range of 4.2~4.4 *μ*m. This is due to the characteristic absorption of CO_2_ and H_2_O in air (Figure 3). At the wavelength of 400~1000 nm and 2.5~15.5 *μ*m, the max of SI was at 5 min of MS burning. With increased burning time, the SI of MSCS decreased gradually, but the variation was not significant (Table 1).

### 3.4. Analysis of Temperature, BP, and SaO_2_ of BL23 Acupoint Skin

**Figure 4:**
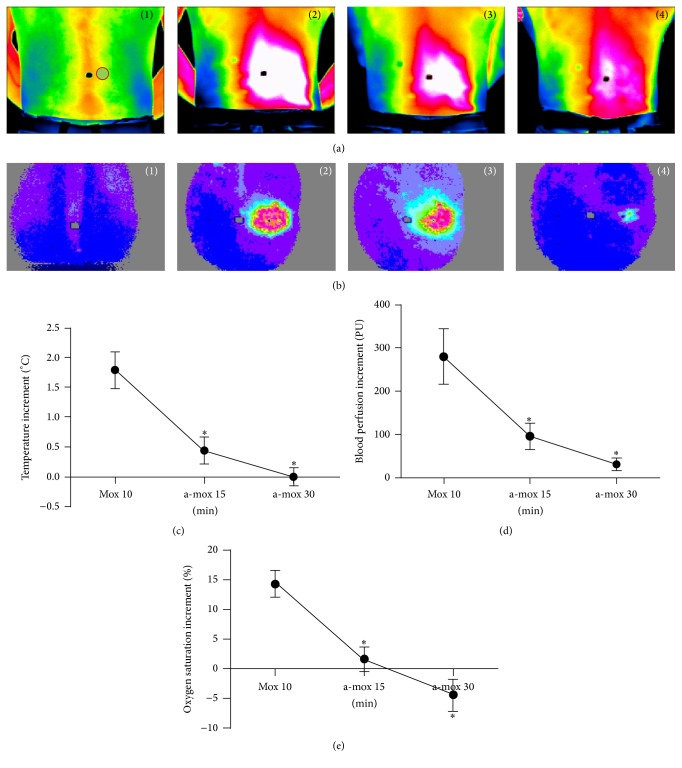
Changes of temperature, blood perfusion (BP), and oxygen saturation (SaO_2_) in BL23 acupoint skin before and after MS moxibustion. (a) Visualization of infrared thermal imaging for right BL23; (a-1) before moxibustion, (a-2) at 10 minutes after moxibustion, (a-3) at 15 minutes after stopping moxibustion, and (a-4) at 30 minutes after stopping moxibustion. The black spot indicated spine. The red circle indicated the location of right BL23. (b) Visualization of laser speckle perfusion imaging (LSPI) for right BL23; (b-1) before moxibustion, (b-2) at 10 minutes after moxibustion, (b-3) at 15 minutes after stopping moxibustion, and (b-4) at 30 minutes after stopping moxibustion. (c) Quantification of temperature increment among groups. (d) Quantification of BP increment among groups. (e) Quantification of SaO_2_ increment among groups. ^*∗*^ *P* < 0.05 versus mox. for 10 min. Data was the difference between BL23 BP of each time point and before moxibustion which are $\bar{x}\pm SE$ (one-way ANOVA with LSD's two groups comparison test).

After MS moxibustion, yellow and litter red areas were displayed on IRT images and LSP images of BL23 acupoint, indicating that the temperature and BP of BL23 acupoint skin both obviously increased (Figures 4(a) and 4(b)). After a period of stopping moxibustion, the color of IRT images and LSP images of BL23 was more and more dark, indicating that the temperature and BP of BL23 gradually decreased. The temperature and BP increments of BL23 at 10 minutes after moxibustion both were significantly higher than those after stopping moxibustion after 15 minutes and moxibustion after 30 minutes too (*P* < 0.05) (Figures 4(c) and 4(d)). SaO_2_ change of BL23 acupoint skin was the same as the temperature and BP. SaO_2_ increment of BL23 was obviously higher compared to that after stopping moxibustion time points (*P* < 0.05). Then SaO_2_ increment declined gradually after stopping moxibustion (Figure 4(e)).

## 4. Discussion

Moxibustion provides very good healthcare and treatment effects. In recent years, researchers have conducted many studies to investigate the mechanism of moxibustion by looking at their clinical, biological, and physical effects. Using HSI and FTIR, the present study revealed the SC of MS during its burning at the wavelengths of 400~1000 nm and 2.5~15.5 *μ*m.

In this study, it was observed that, at the wavelength of 400~1000 nm, the spectrum was distributed at 600~1000 nm, and it ascended at 600~860 nm, descended at 860~1000 nm, and reached its peak at approximately 860 nm. At the wavelength of 2.5~15.5 *μ*m, the spectrum was mainly distributed at 2.7~4.2 *μ*m; the peak position was in the range of 3.0~4.0 *μ*m, and the peak position was approximately 3.5 *μ*m. The results indicate that the radiation spectra of MS include red, near infrared, and far infrared wavelengths, among which the near and far infrared wavelengths are the main components.

Under certain conditions, the energy of the external photons at the wavelength of 100~1000 nm absorbed by molecules of organisms can excite biological chemical reactions, that is, photochemical effects. Among these, the amounts of red and near infrared (600~1000 nm) wavelengths that irradiate an organism can cause changes to cell membrane and inner ultrastructures. A large portion of red light has been absorbed by mitochondria. Consequently, the activity of mitochondria peroxidase has been enhanced, thereby increasing cellular metabolism, promoting the synthesis of proteins and energy metabolism, and causing changes to cellular functions [23]. Additionally, red light can enhance the phagocytosis of leukocytes and transformation of lymphocytes, thereby improving the immune functions of organisms [24]; it can also decrease platelet adhesion, plasma viscosity, and hematocrit, thus promoting tissue repair and accelerating healing [25]. In addition to the aforementioned series of photochemical reactions, the energy of the infrared light absorbed organism can also induce photothermal effects. Water, the prevalent small molecule in organisms, is the main target that interacts with infrared light. Researchers have demonstrated that liquid water can absorb infrared light with a wavelength of approximately 3 *μ*m and 5.7 *μ*m [26]. When infrared light irradiated onto human bodies, its absorption by the water in the cells and blood causes the vibration of O-H and H-O-H bonds in water, thereby converting the infrared light thermal energy to kinetic energy of irregular motions of water molecules and increasing the water temperature. This process subsequently induces cell division and reproduction, promotes the rate of biochemical reactions and enzymatic activity, and simultaneously enhances the structural changes in large biological molecules such as proteins and DNA. Additionally, this process increases the speed of blood flow, thereby improving blood circulation. These series of related biological reactions are the photothermal biological effects of infrared light [27–30]. In this experiment, we found that MS burning can produce red light and a large range of infrared (600~1000 nm) and far infrared radiation (2.7~4.2 *μ*m). According to the discussion above, it was inferred that moderate quantity of moxibustion radiation applied to acupoints or lesions could induce photochemical and photothermal biological effects on organism.

With regard to the biological effects, due to its shorter wavelength and higher energy, the penetration depth of near infrared radiation is ten to tens of times that of far infrared radiation. Consequently, near infrared light can penetrate deep tissues; for example, near infrared light with a wavelength of 0.76~6.6 *μ*m can penetrate 10 mm into the human body, while far infrared can only penetrate 1 mm thick tissues. Near infrared light can penetrate the epidermis, connective tissue, blood vessel, and nervous system and can be absorbed by live tissues. Active substances can be produced in the irradiated tissue after near infrared light irradiating, which can travel to other locations through blood circulation; consequently, the metabolism and heat production of organs are enhanced [31]. It was reported that moxibustion at abdominal acupoints can effectively mitigate peristalsis in IBS patients and improve the pain in inner organs [32]. The moxibustion on the acupoints at the vertex cranii of insomnia patients yields a curing rate of 90.9% for insomnia [33]. Indirect moxibustion can decrease the renal vascular resistance in patients with chronic kidney disease [34]. According to previous studies of moxibustion efficacy and the mechanism of photobiological effect, the relationship between moxibustion effects and SC was analyzed; it was inferred that the more extensive biological effects of moxibustion are intimately linked with the near infrared radiation of moxibustion.

In our previous study, it was observed by HIS technique that the spectral reflex-absorption intensity at the wavelength of 580 nm of Neiguan (PC6) decreased after moxibustion, and the effect of moxibustion was more obvious than acupuncture. It indicated that some factors related to SC of the tissue maybe change after moxibustion and acupuncture, such as functional activities (temperature, microcirculation, material metabolism, etc.) of local tissue and function of the skin tissue structure (texture, sweat gland, and sebaceous gland). And the effect of moxibustion was more obvious [35]. In this study, it was found that the temperature, BP, and SaO_2_ of acupoints obviously increased after MS moxibustion too. These positive effects can continue for some time after stopping moxibustion. Indeed, moxibustion radiation could promote metabolism and microcirculation. However, red, near, and far infrared radiation act on body and can cause photobiological effects, such as increasing microcirculation, promoting metabolism, and improving immune function. In conclusion, it was considered that the SC of moxibustion should be one important material base of moxibustion biological effect. Therefore, it is very significant to study radiation properties of moxibustion for investigating mechanism of moxibustion therapeutic effects.

In this study, it was found that the RRI from the pure MS exhibited a correlation with burning time. But the spectral intensity variation was not significant. This phenomenon is most likely due to the material properties of MS. Because moxa is a plant, the density of the MS compressed into moxa is not uniform. During burning, the energy released is correspondingly nonuniform; additionally, the ashes produced from the static burning that covers the ignition point also affect the radiation of energy.

We obtained the SC of pure MS burning in this experiment. However, what is the share of the photochemical and photothermal effects from the moxibustion-produced radiation of red and of near and far infrared lights in the biological effects of moxibustion? Further studies will be done to investigate the photobiological effects of moxibustion.

## 5. Conclusions

In this study, the new methods of HSI and FTIR were first applied to observe and analyze spectral images of MS moxibustion, accepting SC at wavelengths of 400~1000 nm and 2.5~15.5 *μ*m of MS burning that include red, near infrared, and far infrared light, among which the near and far infrared wavelengths are the main components. It was observed that the temperature, BP, and SaO_2_ of acupoint skin increased after moxibustion too. According to mechanism of photobiological effect and moxibustion biological effect, it was inferred that moxibustion effects were intimately linked with SC of moxibustion. It also provided new data and means for physical properties of moxibustion research.

## Figures and Tables

**Table 1:** The spectrum of MS burning at a wavelength of 400~1000 nm at different time points (*n* = 3).

	Range of spectrum (nm)	Peak of spectrum (nm)
5 min	750–980	860
10 min	755–960	870
15 min	740–980	850
20 min	740–975	880
25 min	740–980	860

## References

[1] Daffara C., Pampaloni E., Pezzati L., Barucci M., Fontana R. (2010). Scanning multispectral IR reflectography SMIRR: an advanced tool for art diagnostics. *Accounts of Chemical Research*.

[2] Isaksson T., Tøgersen G., Iversen A., Hildrum K. I. (1995). Non-destructive determination of fat, moisture and protein in salmon fillets by use of near-infrared diffuse spectroscopy. *Journal of the Science of Food and Agriculture*.

[3] Woo Y.-A., Cho C.-H., Kim H.-J., Yang J.-S., Seong K.-Y. (2002). Classification of cultivation area of ginseng by near infrared spectroscopy and ICP-AES. *Microchemical Journal*.

[4] Ozaki Y. (2012). Near-infrared spectroscopy-its versatility in analytical chemistry. *Analytical Sciences*.

[5] Zhigang R., Bin L. (2011). The principle and application of near-infrared spectroscopy for the chinese herbalmedicines. *Chinese Journal of Pharmaceutical Analysis*.

[6] Shah N., Cerussi A., Eker C. (2001). Noninvasive functional optical spectroscopy of human breast tissue. *Proceedings of the National Academy of Sciences of the United States of America*.

[7] Siddiqi A. M., Li H., Faruque F. (2008). Use of hyperspectral imaging to distinguish normal, precancerous, and cancerous cells. *Cancer Cytopathology*.

[8] Roblyer D., Kurachi C., Gillenwater A. M., Richards-Kortum R. In vivo fluorescence hyperspectral imaging of oral neoplasia.

[9] Chin J. A., Wang E. C., Kibbe M. R. (2011). Evaluation of hyperspectral technology for assessing the presence and severity of peripheral artery disease. *Journal of Vascular Surgery*.

[10] Zuzak K. J., Naik S. C., Alexandrakis G., Hawkins D., Behbehani K., Livingston E. (2008). Intraoperative bile duct visualization using near-infrared hyperspectral video imaging. *The American Journal of Surgery*.

[11] Guanghong D., Xueyong S., Junhao C., Zhiming H., Wei Y. (2002). Observation on the characters of the infrared radiation spectrum of acupoints and four types of moxibustion in the human body. *Zhenciyanjiu*.

[12] Jiang X.-H., Liu H.-P., Guo Z.-Y., Meng Y.-Y., Zeng C.-C., Liu S.-H. (2010). Comparative study of reflectance spectroscopy of women's acupoints around menstruation. *Spectroscopy and Spectral Analysis*.

[13] Zhang J.-B., Wang L.-L., Wu H.-G. (2012). Theory study: warming-dredging and warming-reinforcing of moxibustion. *Zhongguo Zhen Jiu*.

[14] Sun Q., Sun Z.-R., Zhang Q.-H., Wang D., Yue J.-H. (2014). Effect of moxibustion on vascular endothelial cell and expression of vascular endothelial growth factor in rats with cutaneous wound. *Zhongguo Zhen Jiu*.

[15] Peng L., Liu M., Chang X. R. (2014). Role of the nucleus tractus solitarii in the protection of pre-moxibustion on gastric mucosal lesions. *Neural Regeneration Research*.

[16] Xiong J., Liu Z., Chen R., Xie D., Chi Z., Zhang B. (2014). Effectiveness and safety of heat-sensitive moxibustion on bronchial asthma: a meta-analysis of randomized control trials. *Journal of Traditional Chinese Medicine*.

[17] Han Y., Ma T.-M., Lu M.-L., Ren L., Ma X.-D., Bai Z.-H. (2014). Role of moxibustion in inflammatory responses during treatment of rat ulcerative colitis. *World Journal of Gastroenterology*.

[18] Hong Z. G., Guo J. Y., Yin X. F., Wu H. G., Dou C. Z. (2013). Analysis of free radicals in moxibustion smoke. *Chinese Journal of Pharmaceutical Analysis*.

[19] Hong Z. G., Nong Y. Y., Jiang D. (2009). Analysis of chemical composition of combustion products of Artemisia. *Zhongguo Zhen Jiu*.

[20] Zhang H. L., Cheng S. T., Liu Y. P. (1999). Analysis of Chinese moxibustion in visible and infrared spectra in process of clinical application. *Spectroscopy and Spectral Analysis*.

[21] Boldrini B., Kessler W., Rebnera K., Kessler R. W. (2012). Hyperspectral imaging: a review of best practice, performance and pitfalls for in-line and on-line applications. *Journal of Near Infrared Spectroscopy*.

[22] Lu H.-S., Xu H.-R., Ying Y.-B., Fu X.-P., Yu H.-Y., Tian H.-Q. (2006). Application Fourier transform near infrared spectrometer in rapid estimation of soluble solids content of intact citrus fruits. *Journal of Zhejiang University SCIENCE B*.

[23] Karu T. I., Pyatibrat L. V., Kalendo G. S. (2004). Photobiological modulation of cell attachment via cytochrome c oxidase. *Photochemical & Photobiological Sciences*.

[24] Muili K. A., Gopalakrishnan S., Meyer S. L., Eells J. T., Lyons J.-A. (2012). Amelioration of experimental autoimmune encephalomyelitis in C57BL/6 mice by photobiomodulation induced by 670 nm light. *PLoS ONE*.

[25] Melancon M. P., Elliott A. M., Shetty A., Huang Q., Stafford R. J., Li C. (2011). Near-infrared light modulated photothermal effect increases vascular perfusion and enhances polymeric drug delivery. *Journal of Controlled Release*.

[26] Habershon S., Fanourgakis G. S., Manolopoulos D. E. (2008). Comparison of path integral molecular dynamics methods for the infrared absorption spectrum of liquid water. *Journal of Chemical Physics*.

[27] AlGhamdi K. M., Kumar A., Moussa N. A. (2012). Low-level laser therapy: a useful technique for enhancing the proliferation of various cultured cells. *Lasers in Medical Science*.

[28] Zecha J. A., Raber-Durlacher J. E., Nair R. G. (2016). Low-level laser therapy/photobiomodulation in the management of side effects of chemoradiation therapy in head and neck cancer: part 2: proposed applications and treatment protocols. *Supportive Care in Cancer*.

[29] de Vree W. J., Fontijne-Dorsman A. N. R. D., Koster J. F., Sluiter W. (1996). Photodynamic treatment of human endothelial cells promotes the adherence of neutrophils in vitro. *British Journal of Cancer*.

[30] Desmet K. D., Paz D. A., Corry J. J. (2006). Clinical and experimental applications of NIR—LED photobiomodulation. *Photomedicine and Laser Surgery*.

[31] Kim H. P. (2014). Lightening up light therapy: activation of retrograde signaling pathway by photobiomodulation. *Biomolecules and Therapeutics*.

[32] Huang R., Zhao J., Wu L. (2014). Mechanisms underlying the analgesic effect of moxibustion on visceral pain in irritable bowel syndrome: a review. *Evidence-Based Complementary and Alternative Medicine*.

[33] Ju Y.-L., Chi X., Liu J.-X. (2009). Forty cases of insomnia treated by suspended moxibustion at Baihui (GV 20). *Journal of Traditional Chinese Medicine*.

[34] Matsumoto-Miyazaki J., Miyazaki N., Murata I. (2016). Traditional thermal therapy with indirect moxibustion decreases renal arterial resistive index in patients with chronic kidney disease. *The Journal of Alternative and Complementary Medicine*.

[35] Zhang D., Li Y.-L., Wang S.-Y., Bai X.-D., Song X.-J., Li S.-Y. (2014). A pilot study on effects of acupuncture and moxibustion by hyperspectral imaging technique. *Evidence-Based Complementary and Alternative Medicine*.

